# Assessing Community-Level and Single-Species Models Predictions of Species Distributions and Assemblage Composition after 25 Years of Land Cover Change

**DOI:** 10.1371/journal.pone.0054179

**Published:** 2013-01-17

**Authors:** Sébastien Bonthoux, Andrés Baselga, Gérard Balent

**Affiliations:** 1 INRA, UMR1201 DYNAFOR, Castanet-Tolosan, France; 2 UMR CITERES 7324, ENSNP, Blois, France; 3 Departamento de Zoología, Facultad de Biología, Universidad de Santiago de Compostela, Santiago de Compostela, Spain; Institute of Botany, Czech Academy of Sciences, Czech Republic

## Abstract

To predict the impact of environmental change on species distributions, it has been hypothesized that community-level models could give some benefits compared to species-level models. In this study we have assessed the performance of these two approaches. We surveyed 256 bird communities in an agricultural landscape in southwest France at the same locations in 1982 and 2007. We compared the ability of CQO (canonical quadratic ordination; a method of community-level GLM) and GLMs (generalized linear models) to i) explain species distributions in 1982 and ii) predict species distributions, community composition and species richness in 2007, after land cover change. Our results show that models accounting for shared patterns between species (CQO) slightly better explain the distribution of rare species than models that ignore them (GLMs). Conversely, the predictive performances were better for GLMs than for CQO. At the assemblage level, both CQO and GLMs overestimated species richness, compared with that actually observed in 2007, and projected community composition was only moderately similar to that observed in 2007. Species richness projections tended to be more accurate in sites where land cover change was more marked. In contrast, the composition projections tended to be less accurate in those sites. Both modelling approaches showed a similar but limited ability to predict species distribution and assemblage composition under conditions of land cover change. Our study supports the idea that our community-level model can improve understanding of rare species patterns but that species-level models can provide slightly more accurate predictions of species distributions. At the community level, the similar performance of both approaches for predicting patterns of assemblage variation suggests that species tend to respond individualistically or, alternatively, that our community model was unable to effectively account for the emergent community patterns.

## Introduction

The distributions of many species and communities are showing rapid changes in the face of habitat and climate change [1]–[4]. Predicting where and under which scenarios changes in species compositions are likely to occur is a major challenge in fundamental and applied ecology [5]. Attempts to predict the impact of global change on communities of species are usually made by developing models based on statistical relationships between species and their environment [5]–[7].

The most popular strategy for providing maps of actual or potential species distributions has been to model distributions of individual species one at a time [5]. This approach assumes that species respond individualistically to environmental changes. However, the distribution of species can potentially be influenced by the distribution of other taxa, so models should better take into account positive and negative associations between species [8], especially on finer scales of analysis. It has been suggested that community-level modelling [9] could confer significant benefits for applications involving very large numbers of species, particularly where a sizeable proportion of those species are rarely recorded in the dataset. Unlike species-level modelling, for which species with too little data are usually excluded from further analysis (for statistical reasons), many community-level modelling strategies make use of all available data across all species, regardless of the number of records per species [9]. Moreover, this approach takes into account the patterns of co-occurrence of species in the statistical analysis, assuming that interspecific associations are indirectly accounted for by patterns of co-occurrence (or co-exclusion). Although some studies have compared community-level models with individual distribution models, it is not clear whether community-level models outperform individual models. Elith & Leathwick [10] found that community-level models generally performed better for plants, birds, mammals and reptiles at a finer spatial resolution (≤1 km). In contrast, Baselga & Araújo [11] found that individual models had a greater ability to predict the occurrence of 119 European tree species at a 50 km grid square resolution. More recently, Chapman & Purse [12] found that community level models were slightly less accurate than single-species models, but that they offered a highly simplified way of modelling spatial patterns in British plant community. None of these earlier studies compared the performance of single-species and community models using independent validation data collected at a different time. However, species distributions are the result of dynamic processes in which the temporal dimension cannot be overlooked [13]. Using data collected at another date is an independent validation which is considered to be the best option for measuring the ability of models to predict new situations [5], [6], [14]–[16]. Indeed, several studies have shown that using non-independent validation as cross validation can lead to an overestimation of the predictive capabilities compared with independent validation [17]–[19] and potentially to a poor application of models in conservation planning. On fine scales, land use plays a major role in species distributions [20], [21]. Land use changes are obviously related to human actions, especially in agricultural landscapes where intensification of agricultural practices has led to a sharp decline in natural land cover and a homogenization of landscapes in Europe [22], [23]. Unfortunately, it is often difficult to obtain information on past land use and, in practice, very few studies have explicitly assessed the predictive performance of distribution models in a context of land use change (but see [24], [25]).

In this study, we compare the ability of community-level and single-species models to provide accurate predictions of species distributions in a context of land cover change. We attempt to answer this question using distributional bird data recorded in southwest France in two different years, 1982 and 2007. On fine spatial scales (e.g. territory scale) biotic interactions between birds can be strong. During the breeding season, individuals have a strong conspecific and interspecific competition to defend their territory from other individuals [26]–[28]. Moreover several studies have highlighted potential associations between species in bird assemblages using analyses of co-occurrence patterns [29], [30]. We thus hypothesize that community-level model can be substantially more accurate than single-level models to predict bird assemblage patterns. Specifically, we examine 1) whether the explanatory capacity and the accuracy of species distribution predictions based on land cover variables differ between community-level and single-species models, 2) whether differences in predictive accuracy between community-level and single-species models may be associated with species number of occurrences, and 3) whether the predictive accuracy of species richness and composition differs between community-level and single-species models and, if so, whether the amplitude of landscape change can explain these differences.

## Methods

### Ethics Statement

Approval for this work and for the field campaigns was done in consultation with all farmers of the study site.

### Study Site

The study site lies between the Garonne and Gers rivers, in southwest France (43°16′28″ N, 0°51′11″ E, WGS 1984) and is part (approximately 260 km^2^) of the “Coteaux de Gascogne” Long Term Ecological Research site (LTER_EU_FR_003). The area is hilly (altitude 200–400 m) and dissected by north-south valleys, within a sub-Atlantic climate subject to both Mediterranean and mountain influences. Forest cover is fragmented, and currently covers some 15% of the area. Woodlands are dominated by *Quercus robur* and *Quercus pubescens*. Dominant non-forest land-use modalities consist of a combination of crops (including maize, oilseed rape, sorghum, sunflower and forage crop), grasslands, hedges and small woodlands. Grasslands are not reseeded for at least five years (in accordance with the Common Agricultural Policy), and sometimes several decades. They are grazed and/or mowed. Hedges are mostly composed of shrubs and sometimes trees, which on average are two metres high.

### Biological Data and Environmental Predictors

We used a set of 256 point counts, each recorded twice, in 1982 and 2007. In 1982, the point counts were settled in a stratified design representing the diversity of land-use types (Figure S1). The point counts were separated at least by 250 m. This distance is greater than the home range size of most of the studied species during the breeding period (usually less than 2 ha, [31]). In 1982 and 2007, the presence-absence of each bird species was recorded within a 125 m radius around each point, during 20-minute periods. Bonthoux & Balent [32] have shown that the count duration (from 5 to 20 minutes) does not impact the explanatory and predictive performances of species distribution models. The counts were conducted between 6∶00 and 11∶00 in the morning during the birds’ vocal activity peak. Very windy and rainy conditions were avoided in order to limit any detectability problems. The dataset consisted of species presence-absence records to limit biases associated with abundance data. We excluded raptor species from the analysis as the point count method is not suited to their large home range, and urban species (e.g. house sparrow, swallows) because the point-count distribution was not stratified in such a way as to obtain a gradient of urbanisation. The final dataset comprised 35 farmland and woodland species (Table S1). In 1982, the rarest species was *Upupa epops* (present in 6 point counts) and the most common species was *Sylvia atricapilla* (present in 192 point counts).

**Figure 1:**
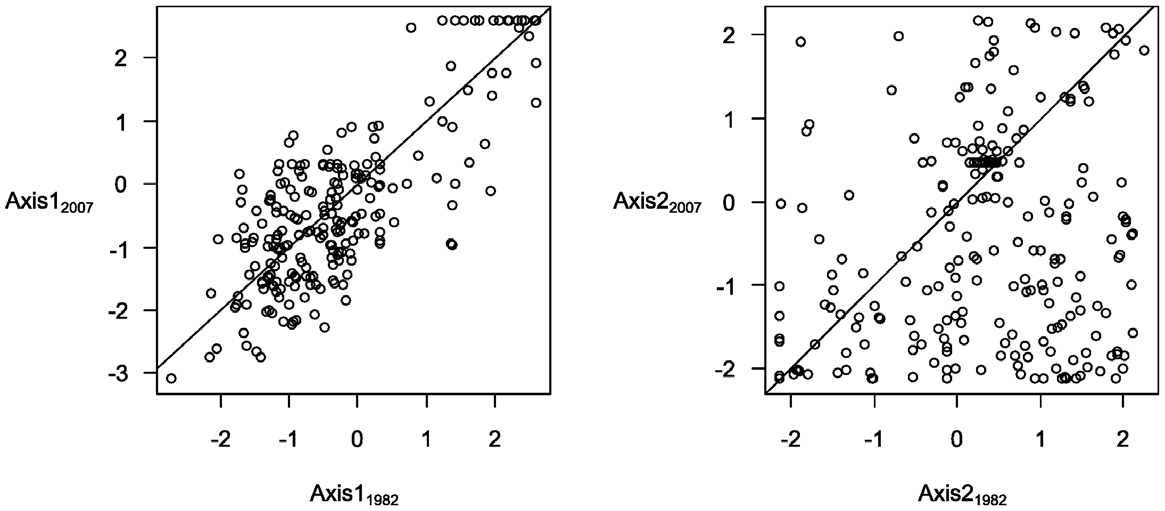
Position of the stations along the Axis 1 and Axis 2 of the PCA in 1982 and 2007. The equation of the line is y = x.

For this study, we were limited to the use of two environmental predictor variables (see below). In order to select two variables, we started with six landscape variables that were shown to be relevant to explain bird distributions [33]: percentage of woodland, fallow, permanent grassland, crops, length of hedge and a Shannon heterogeneity index based on the percentage of each land cover variable. To quantify these variables we used aerial photographs dating from 1979, and the BDOrtho© orthorectified digital photograph database dating from 2006 (French National Geographical Institute, IGN), the landscape data closest to the years in which the bird censuses were taken (1982 and 2007). We digitized land-use variables in a 125 m buffer centred on each point count using ArcGIS 9.2 (Environmental Systems Research Institute, Inc.) and checked the interpretation of aerial photographs with field observations made during the bird censuses. These six variables were submitted to a Principal Components Analysis (PCA) based on 1982 and 2007 data (N = 512). The first two components accounted for 59% of the variance. The first axis was an opening landscape gradient from wooded areas to open areas with hedge. The second axis was a gradient from simple landscapes with crops to heterogeneous landscapes with permanent grasslands (Table 1). There was no significant change along Axis 1 between 1982 and 2007 (paired *t* test, *t* = 1.45, p = 0.10). In contrast, due to the intensification of agricultural practices in this region [34], there was a significant increase along Axis 2 between 1982 and 2007 of the percentage of simple landscapes with crops at the expense of heterogeneous landscapes with grasslands (*t* = 7.70, p<0.001) (Figure 1).

**Table 1:** Principal components of environmental variables.

	Axis 1	Axis 2
Wood (%)	**0.88**	0.24
Fallow (%)	0.04	0.03
Grassland (%)	−0.56	**0.70**
Hedge (m)	**−0.78**	0.22
Crop (%)	−0.38	**−0.90**
Shannon index	−0.55	**0.70**

Values in bold represent factor loadings contributing the most to each axis.

### Selection of Single-species and Community-level Models Used

In accordance with Baselga & Araújo [11], we selected two modelling procedures (single-species vs. community-level) that are directly comparable because differences between their ouputs are univocally attributable to their single-species or community-level nature: GLM (Generalized Linear Model) and CQO (Canonical Quadratic Ordination) [35]. CQO, like other ordination techniques, explicitly accounts for co-occurrence and exclusion patterns while enabling projections of the distribution of each species. This community-level method can be viewed as a system of simultaneous regression equations (simultaneous GLMs) to integrate species occurrence/exclusion information (see [35] for mathematical details of CQO). It is thus a more advanced alternative compared with more familiar CCA (Canonical Correspondence Analysis), because CQO does not make the unrealistic assumptions made by CCA (for example equal environmental tolerances and maxima for all species) and allows the projection of the species responses as a function of environmental predictors. CQO is fitted with GLM and assumes quadratic responses of species to predictor environmental variables. We are conscious that the shapes of species responses to environmental gradients can be very varied but several studies have shown that the quadratic shape is well suited to relationships between birds and landscape components [36], [37].

As proposed by Baselga & Araújo [11], we identified two orthogonal variables (with PCA, see above) and fitted these variables to 1) single-species distribution models (referred to as GLM throughout the text) and 2) a community model simultaneously including all the species in a Rank-2 CQO model (referred to as CQO throughout the text). CQO identifies a set of orthogonal latent variables from a combination of environmental variables. By using just two orthogonal variables we ensured that the latent variables were equivalent to the individual variables entering the model. With this procedure we ensured that differences between the Rank-2 CQO and GLM models could only be attributable to the co-occurrence/exclusion patterns.

### Model Calibration

Data from 1982 were used to fit the CQO and GLM. Species distributions were modelled individually using GLM with binomial errors, logit link and quadratic functions (y = x+x^2^). Response variables were presence-absence records and predictor variables were the two axes of the PCA. No variable selection was implemented and the quadratic linear terms of the two axes were automatically included in models for all species in order to allow full compatibility with CQO. For the community-level model, a Rank-2 CQO was fitted to the occurrence of the 35 species, using binomial errors, logit link and the two axes of the PCA as predictor variables.

We found no evidence of spatial autocorrelation between the models’ residuals based on non parametric spline correlograms (‘ncf’ package), indicating that non-spatial statistical models were appropriate [38].

### Model Validation

First, to evaluate the models’ explanatory performances, we calculated the percentage of explained deviance (% D^2^) for each species. Then, we used an independent validation which is the best approach to evaluate the predictive performance of species distribution models [5], [15]. We calibrated models on the entire 1982 dataset and compared the predictive performances of CQO and GLM using data from 2007. We are aware that some degree of dependence exists between the two dataset, as they were recorded in the same area at two time periods. However, in practical terms, we assume that these two datasets are independent events, as the samplings were carried out 25 years apart. We tested agreement between observed and predicted distributions by calculating four measures of accuracy: the AUC (area under the curve) of ROC (receiver operating characteristic) curve, the sensitivity, the specificity and the Brier index. The reliability of predictions is considered null for AUC values <0.5, poor when the AUC values are between 0.5 and 0.7, correct for values between 0.7 and 0.8 and good when they are >0.8 [39]. Compared with AUC, which is threshold-independent, the sensitivity and the specificity are calculated from the confusion matrix. We used the prevalence (i.e. the number of presences divided by the total number of point counts) of each species in the calibration set as a threshold for converting the predicted probabilities into presence-absence scores [40]. Sensitivity is the probability that the model will correctly classify a presence, and specificity is the probability that the model will correctly classify an absence. The Brier index, which is equivalent to RMSE for abundance data, is the root mean square error between the observed and the predicted values. The reliability of predictions decreases when Brier values increase. The comparison between the explanatory and predictive performances of GLM and CQO for the five criteria was made using a Wilcoxon paired test. Finally, we used Spearman correlation tests to assess a possible link between the differences of performance of each modelling approach for the five criteria (for example %D^2^_CQO_ − %D^2^_GLM_) and the number of occurrences of each species in 1982.

### Projected Assemblages and Land Cover Change

The GLM and CQO models fitted to the entire 1982 dataset were used to project each species’ occurrence probability under 2007 environmental conditions. There is currently a debate on how to model species richness using stacking predictions based on individual species distributions [41]. The approach based on summing binary maps tends to yield a strong and constant overprediction of species richness but can predict individual species and thus community composition. Alternatively the other two approaches - summed binomial trails based on predicted probabilities and summed predicted probabilities - do not overpredict species richness overall, but overestimate species-poor sites while species-rich sites are underestimated, and reproduce species richness patterns badly along an environmental gradient [41]. Moreover they do not provide a single unequivocal final species composition [41]. Because our goal was simultaneously to project species richness and composition, we used an approach based on summing binary maps.

Species richness was computed for each modelling approach (S_GLM_, S_CQO_) as the sum of all presences projected by GLM and CQO and for 2007 real observations (S_OBS_) as the sum of all presences observed in each station. The difference between both model values (ΔS_MODELS_ = S_CQO_ **-** S_GLM_) and the differences between projected and observed richness (ΔS_CQO_ = S_CQO_ **-** S_OBS_, ΔS_GLM_ = S_GLM_ **-** S_OBS_) were regressed against changes in environmental predictors (ΔAxis1 =  Axis1_2007_– Axis1_1982_ and ΔAxis2 =  Axis2_2007_– Axis2_1982_) to assess environmental trends in models.

To examine differences in species composition between models (β_MODELS_) and between models and observations (β_CQO,_ β_GLM_) we used the Simpson index of dissimilarity [42], [43]. The Simpson index is a measure of differences in composition independent of the differences in richness between samples [43], [44]. We then assessed the link between these compositional dissimilarity indices and the two environmental predictors ΔAxis1 and ΔAxis2.

All the above mentioned statistical analyses were carried out in R (R Development Core Team 2009) using libraries VGAM and PresenceAbsence.

## Results

**Figure 2:**
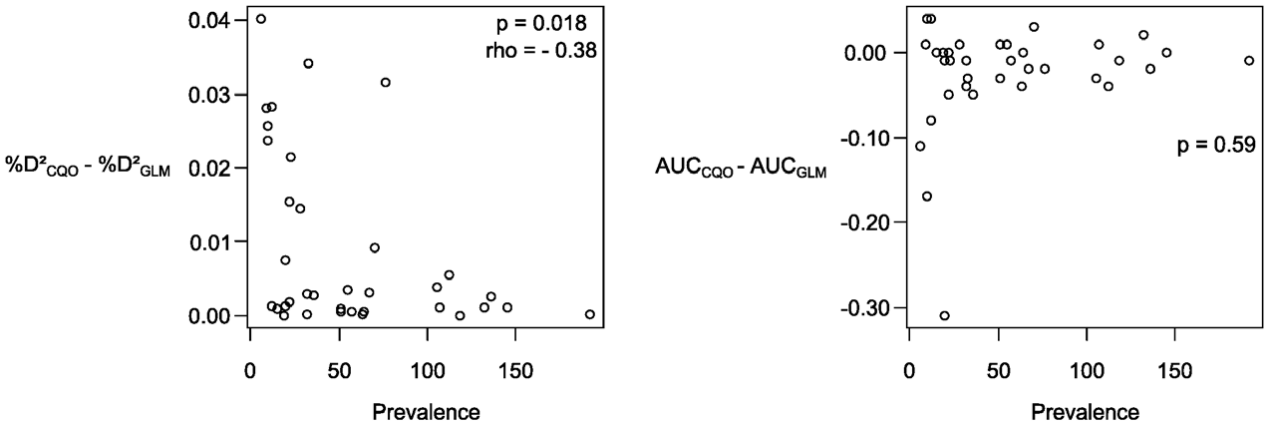
Relationships between the differences in explained deviance (%D^2^) and AUC between CQO and GLM and the number of occurrences of each species (Spearman rank correlation).

The explanatory and predictive performances of each modelling approach (CQO and GLM) are summarized in Table 2. The percentage of explained deviance was significantly and moderately higher for CQO than for GLMs. Conversely, AUC and specificity values were significantly higher for GLMs than for CQO. Brier values were significantly lower for GLMs than for CQO, indicating that the reliability of species distribution predictions was better for GLMs based on this criterion. Sensivity values tended to be higher for CQO than for GLMs but these differences were not significant. There was a significant negative correlation between the difference in explanatory performance (% D^2^) of CQO and GLM with the number of occurrences of each species in 1982 (Spearman rank correlation, p = - 0.38, p = 0.018) (Figure 2), but no significant correlation between the difference in predictive performance of CQO and GLM and the number of occurrences.

**Table 2:** Explanatory and predictive performances expressed by five criteria for CQO and GLM.

	D^2^			Sensitivity			Specificity		
	mean (SD)	range	p	mean (SD)	range	p	mean (SD)	range	p
**CQO**	0.17 (0.09)	0.03–0.38	<0.001	0.75 (0.20)	0.22–1	0.07	0.66 (0.18)	0.30–0.95	0.03
**GLM**	0.16 (0.09)	0.03–0.35		0.71 (0.24)	0–1		0.72 (0.18)	0.36–1	
	**AUC**			**Brier**					
	mean (SD)	range	p	mean (SD)	range	p			
**CQO**	0.71 (0.09)	0.51–0.88	0.008	0.39 (0.13)	0.17–0.67	<0.001			
**GLM**	0.73 (0.10)	0.52–0.88		0.33 (0.11)	0.12–0.50				

We did a Wilcoxon paired test to compare these performances between the two modelling approaches.

**Figure 3:**
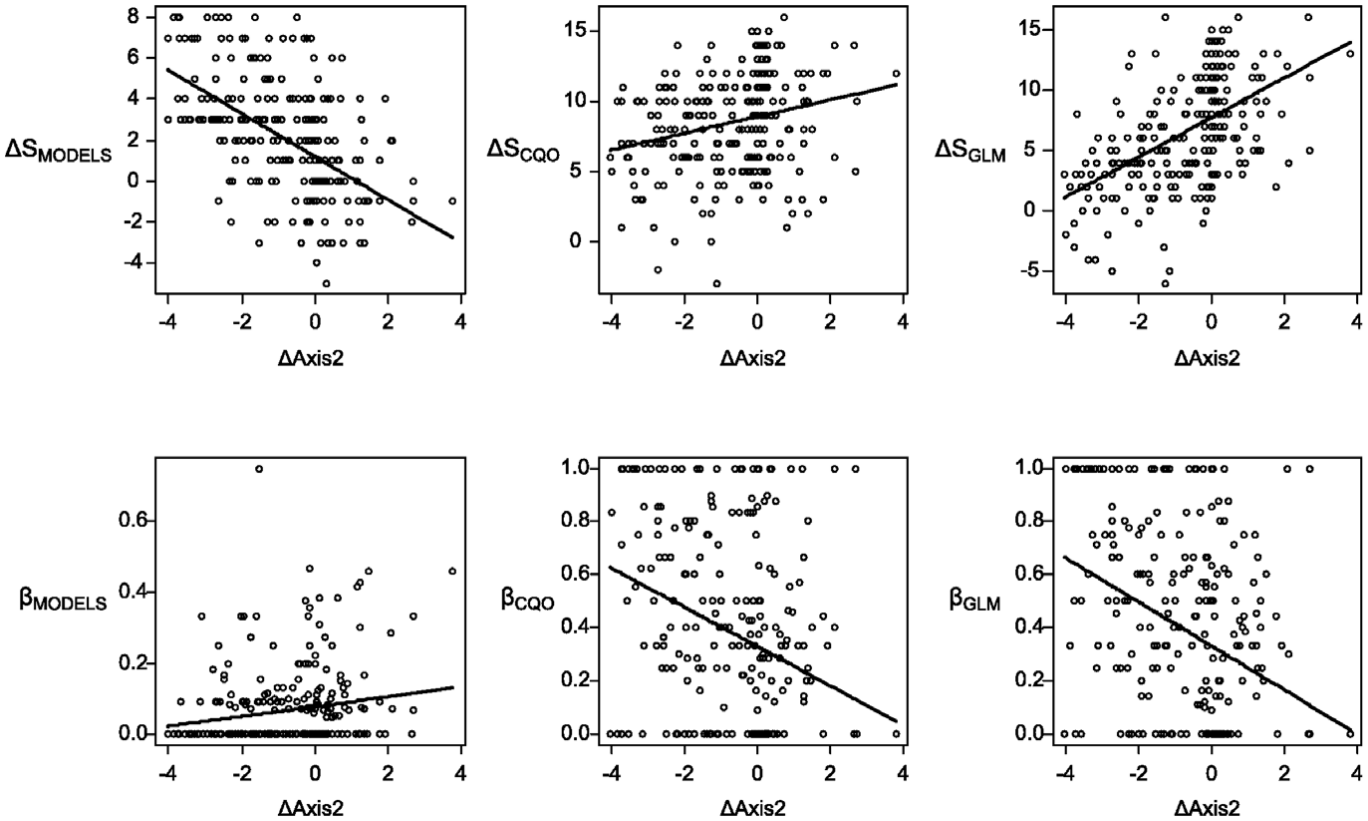
Differences in species richness and species composition projected by both approaches CQO and GLM under 2007 environmental conditions and differences in species richness and species composition projected by CQO, GLM and 2007 real observations (respectively ΔS_MODELS_, β_MODELS_ and ΔS_CQO_, ΔS_GLM_, β_CQO_, β_GLM_). These differences were correlated with changes in environmental predictors between 1982 and 2007.

Species richness projected for 2007 with CQO (S_CQO_) and GLM (S_GLM_) were significantly higher than species richness observed in 2007 (S_OBS_) (mean (S_CQO_–S_OBS_) = 8.50; SD = 3.45; *t* = 27.49; p<0.001; mean (S_GLM_ - S_OBS_) = 6.57; SD = 4.52; *t* = 16.45; p<0.001). When the two modelling approaches’ richness projections were compared, S_CQO_ was significantly higher than S_GLM_ (mean (S_CQO_ - S_GLM_) = 1.94; SD = 2.80; *t* = 4.54; p<0.001). ΔS_MODELS_ was not significantly related to ΔAxis1 but showed a significant negative relationship with ΔAxis2 (*r* ^2^ = 0.30, p<0.001). ΔS_CQO_ was not significantly related to ΔAxis1 but positively related to ΔAxis2 (*r* ^2^ = 0.08, p<0.001). ΔS_GLM_ was not significantly related to ΔAxis1 but positively related to ΔAxis2 (*r* ^2^ = 0.28, p<0.001) (Figure 3).

Dissimilarity between projected and observed composition in 2007 was moderate (mean β_CQO_ = 0.38; mean β_GLM_ = 0.39) and not significantly different between approaches (*t* = 0.29, p = 0.77). The lack of differences between approaches derived from the fact that the dissimilarity between composition projected by CQO and GLM was small (mean β_MODELS_ = 0.07). β_MODELS_ was not significantly related to ΔAxis1 but showed a significant positive relationship with ΔAxis2 (*r* ^2^ = 0.04, p = 0.005). β_CQO_ was not significantly related to ΔAxis1 but negatively related to ΔAxis2 (*r* ^2^ = 0.08, p<0.001). β_GLM_ was not significantly related to ΔAxis1 but negatively related to ΔAxis2 (*r* ^2^ = 0.11, p<0.001) (Figure 3).

## Discussion

In this study, we assessed the ability of community-level (CQO) and single-species models (GLMs) to predict species distributions, richness and composition under land cover change, using, for the first time truly independent validation data: models were fitted with data obtained in 1982 and validated with data obtained in 2007 (i.e. after land cover change had actually taken place). Our results showed that models accounting for shared patterns of occurrence between species (CQO) explain better the distribution of rare species in the calibration data set than models that ignore shared patterns (GLMs). Despite this, the predictive performance of GLMs was better based on AUC, specificity and Brier values. At the assemblage level, when the predicted distributions of species were combined, both CQO and GLM overestimated the observed species richness, with the overestimation being larger for CQO than for GLM. The difference between observed and projected species richness varied along gradients of land use change, as the tendency of CQO and, even more so, GLM to overestimate richness was lower on sites where crop cover increased between the two dates. CQO and GLM projected very similar community compositions, but in both cases the difference between projected and observed composition was moderate. Contrary to the results for species richness, differences between projected and observed composition were lower in sites where the area dedicated to permanent grasslands increased.

So far, studies of the relative performance of community-level versus species-level models have essentially focused on the predictive abilities of models and very few studies have compared their explanatory abilities. Using multivariate adaptive regression splines (MARS), Leathwick *et al.* [45] found that individual models explain a greater amount of deviance compared with a multispecies model. Guisan *et al.* [46] found that single-species models explain the distribution patterns of trees and shrubs in Nevada much better than the community-level model. However they used very different mathematical models, including GLMs with polynomial terms for single species and CCA (Canonical Correspondence Analysis) that links species and environmental variables with linear relationships, so it is unclear whether differences they found are due to the type of model or to the inclusion of shared distribution patterns. Chapman & Purse [12] used species and community-level approaches based on the same statistical family but they did not compare explanatory performances. The two approaches used in our study to implement individualistic and community analyses were comparable in that they were based on the same regression algorithms and used the predictor variables in the same ways. Under these circumstances, we show that CQO explains species distributions slightly better than GLM, with the difference being greatest for rare species.

In contrast with the previous result on explanatory performance, we found the predictive ability of models for our system (bird species at fine spatial resolution) was lower for CQO than for GLM (based on AUC and Brier values). These results are in agreement with those by Baselga & Araújo [11], who found that GLM provide more accurate projections than CQO for European tree species on large spatial scales. Using different modelling algorithms, the same result was replicated for British plants by Chapman & Purse [12], who found that univariate regression trees and artificial neural networks had higher predictive ability than their multivariate extensions. If the former results could be generalised, the fact that taking into account shared patterns of species induces poor predictive performances might mean that transferability of shared patterns over time is low. In other words, the fact that a higher explanatory performance in CQO does not translate into a higher predictive performance could thus point to a probable overfitting of data by the CQO model caused by the fact that this model accounts for patterns of co-occurrence. Ferrier & Guisan [9] hypothesized that the appropriateness of modelling biodiversity at the community level, as opposed to the species level, is likely to vary depending on the purpose of a given study. Specifically, they hypothesized that community-level models can bring benefits compared with species-level models when rare species are present in the dataset. In our study, the community-level model is better for explaining the patterns of rare species in the calibration dataset, but single-species models are slightly more useful to predict patterns of species distributions in the validation dataset.

We also found that GLM predicted absences (higher specificity) slightly more efficiently than CQO. In contrast, CQO models tended to predict better presences (higher sensitivity) than GLM. This is relevant because reliably predicting species’ presences may be preferred to a good prediction of absences in the context of conservation studies, e.g. when the objective is to choose reserve areas. These results are the opposite of those obtained by Baselga & Araújo [11] for trees. They found that GLM had higher sensitivity than CQO, but that CQO had higher specificity than GLM. These results might indicate that relative performance of GLM and CQO could be case-dependent, although in general terms differences in predictive performance between GLM and CQO seem small in both situations, suggesting that even when co-occurrence patterns can *a priori* be hypothesized to be a highly relevant factor, community-level models do not significantly improve predictive performance, as suggested by present and previous results [11], [12], [47]. Therefore, further research should examine whether shared patterns do not have the previously attributed relevance or whether community-level models fail to account for biotic interactions (even if indirectly).

Both community-level and single-species models overestimated species richness, compared to the richness values actually observed in 2007. Previous contributions have shown that the aggregation of predicted species distributions based on summing binary maps tends to overestimate the true species richness [39], [48].This overestimation could be attributed to the fact that species do not occupy all the sites where the habitat is suitable, i.e. species distributions are not in equilibrium with the environmental conditions [49]–[51]. Despite the fact that both approaches overestimated species richness in 2007, the community-level model (CQO) predicted even higher species richness than the single-species modelling (GLM), as also found for European trees by Baselga & Araújo [11]. This larger overprediction is due to the fact that CQO predict more false presences than GLM. Specifically, CQO predicted higher species richness than GLM in sites where the amount of crops increased. Where landscapes became more cultivated and homogeneous, GLM predicted the presence of species associated with open landscapes (*Alauda arvensis, Emberiza calendra, Sylvia communis, Saxicola torquata*). In these sites, CQO overestimated richness by adding some other species that are not characteristic of cultivated habitats but of heterogeneous landscapes (e.g. *Anthus trivialis, Carduelis cannabina, Emberiza citrinella, Picus viridis*). This result is linked to the fact that the predictive performances of CQO compared with GLM were low for those species. In other words, the effect of co-occurrence patterns makes CQO to overestimate (compared with GLM) the distributions of some species characteristic of heterogeneous landscapes, predicting their presence in cultivated habitats where in fact they were not found. Regarding species composition, the assemblages predicted by both CQO and GLM for the 2007 conditions were moderately different from the observed composition in 2007. Besides, due to the overestimation by CQO described above, assemblages predicted by GLM were often subsets of assemblages predicted by CQO.

Interestingly, the accuracy of species richness and composition predicted by the models differs according to the amplitude and direction of landscape change. In localities where crops cover increased, the species richness predictions tended to be more accurate, whereas the composition predictions tended to be less accurate. Taking into account the fact that observed richness decreases with the increment in crops [33], [52], both results taken together suggest that composition is only more accurately predicted when a high number of species is predicted to be present, and the observed composition is then a subset of the predicted composition. In sites where crops increased, the models’ species richness errors are smaller, probably because both predicted and observed richness are lower. But in these conditions, the predicted composition is very different from what is observed. This means that under these circumstances of marked land cover change, predictive models are not very useful, because even if they predict the species richness accurately, they predict the presence of species that are not actually observed. At the other extreme, in localities where permanent grasslands increased, the models correctly predicted the presence of observed species, but at the cost of predicting many other species that are not actually observed. So, the models identified sites where natural habitats increased or remained as suitable for a high number of species, but not all the species that could potentially live in a given locality are actually observed there. This moderate predictive performance of models may be due to the model structure. Although relevant in terms of management actions, the land cover variables included in the models indirectly reflect species’ resources (e.g. food availability, breeding site). Land cover variables may be correlated differently to resources on both dates leading to difficulties predicting species distributions and community patterns. We just included two landscape variables in models (the two first components of a PCA built with height landscape variables). Including other environmental information (e.g. local vegetation structure, topography) could potentially increase the amount of explained deviance and the accuracy of model predictions. Thus the results of this study and specifically the relative accuracy of alternative modelling approaches could be potentially different given the availability of more environmental data. Another potential reason that could explain these results might be the above-mentioned non-equilibrium of species distributions with environmental conditions [50]. Given that on the geographic scale of this study no major dispersal limitation effects are expected, the lack of equilibrium could be due to stochastic local absences of bird species in environmentally suitable sites. Stochastic events may play a major role for explaining habitat use patterns, especially on small spatial scale [53].

The community-level approach considered in this study takes into account the statistical associations of species along environmental gradients but cannot explicitly model positive and negative interactions between species. Therefore, it would be interesting to compare this kind of community-level models with new promising approaches that consider more explicitly species interactions [54]. For instance, the use of multivariate logistic regressions based on spatial multispecies co-occurrence patterns [55] needs to be explored in a context of environmental change.

A promising line of research is the use of predictive habitat models to forecast conflicts between human activities and biodiversity conservation. This is the case when assessing the impact of land-use changes linked to evolving agricultural practices [56]. This issue means a scale must be found for which the process driving agricultural management matches the ecological processes [57]. On a fine scale (4.9 ha) which is relevant for the application of agri-environment schemes [58], [59], we found that the models only had a moderate ability for projecting species distributions and assemblage patterns. Further studies are therefore needed to find a scale that links the human and ecological processes. In a context of land cover change, SDM are widely used tools for predicting general patterns of species distributions and providing management recommendations. However, our results show that model projections have to be used with caution, especially in situations of marked temporal change in environmental conditions.

It has been suggested that community-level models taking into account co occurrence/exclusion patterns deserve to be used more often, as an alternative or in addition to single-species models [9]. Here, we compared the ability of community-level and single-species models to explain patterns and make accurate predictions under land cover change using independent validation. Our study support the idea that our community-level model (CQO) can be better to understand assemblage patterns composed of rare species. This point is important because many species of conservation interest are rare. In contrast, our results suggest, in line with previous studies, that our species-level models (GLMs) would be better for predicting species distributions. At the community level, the similar performance of both approaches for predicting patterns of assemblage variation suggests that species tend to respond individualistically or, alternatively, that our community model was unable to effectively account for the emergent community patterns.

## Supporting Information

Figure S1 **The study site showing the 256 point counts performed in 1982 and 2007.** In 1982, the point counts were settled in a stratified design of 21clusters representing the diversity of land-use types. The point counts were separated from each other by 250 m in each cluster. Represented land uses are woodlands (black), grasslands (dark grey), crops (light grey), buildings (hatched) and ponds (white) (EuropeanUnion–SOeS, CORINE Land Cover, 2006; this map was not used to calculate land-use percentages in analyses, see “Method”).
(TIF)

Table S1 The number of sites occupied by bird species in 1982 and 2007 (N = 256).
(DOC)
