# Supplementary figures and images for: Assessing Community-Level and Single-Species Models Predictions of Species Distributions and Assemblage Composition after 25 Years of Land Cover Change
Source: PLoS One. 2013 Jan 17;8(1):e54179. doi: 10.1371/journal.pone.0054179 (PMC3547884; doi:10.1371/journal.pone.0054179)

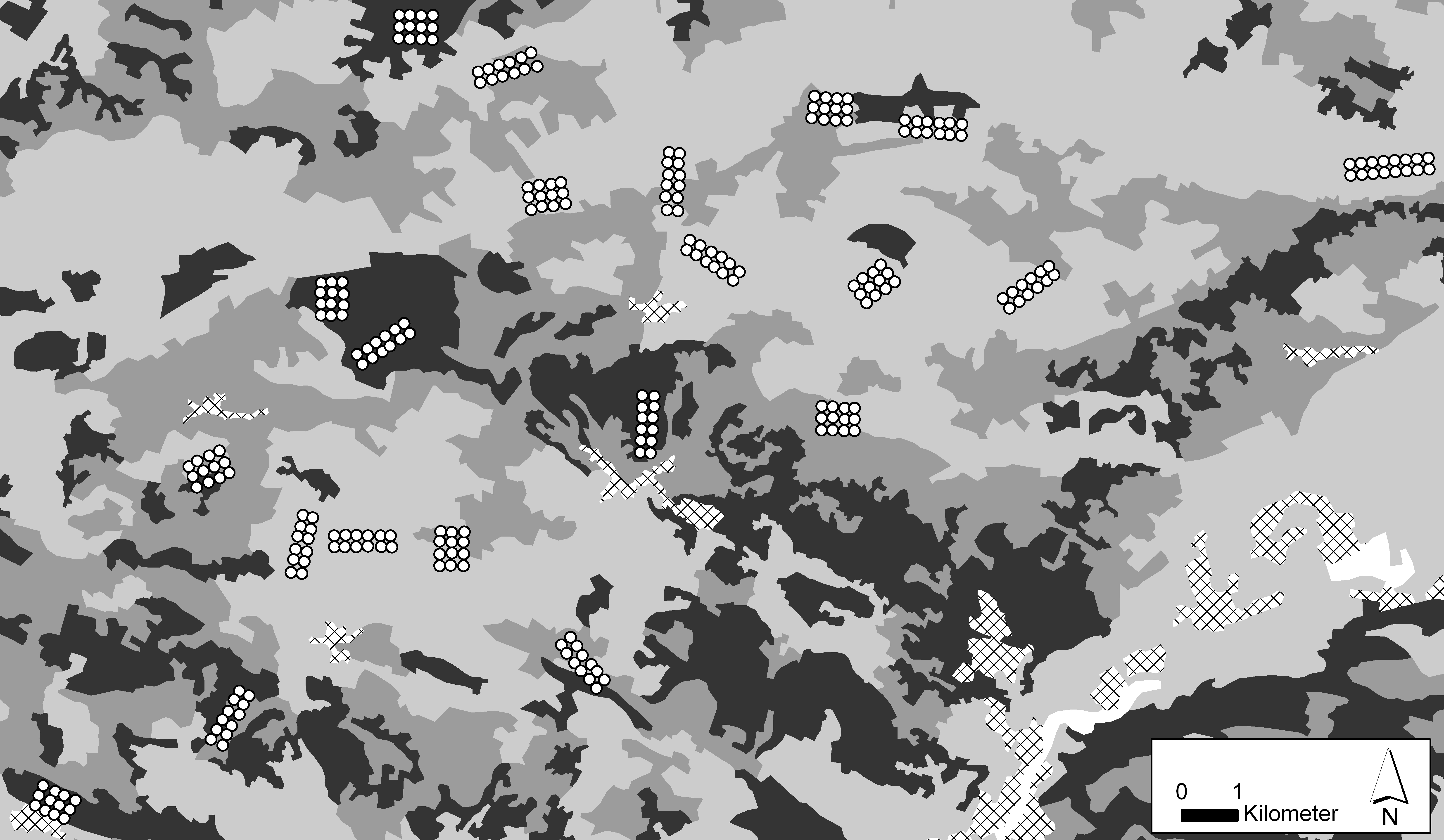

Supplement: Figure S1 — The study site showing the 256 point counts performed in 1982 and 2007. In 1982, the point counts were settled in a stratified design of 21clusters representing the diversity of land-use types. The point counts were separated from each other by 250 m in each cluster. Represented land uses are woodlands (black), grasslands (dark grey), crops (light grey), buildings (hatched) and ponds (white) (EuropeanUnion–SOeS, CORINE Land Cover, 2006; this map was not used to calculate land-use percentages in analyses, see “Method”). (TIF) [file pone.0054179.s001.tif]
