# Supplementary material for: Assessing Community-Level and Single-Species Models Predictions of Species Distributions and Assemblage Composition after 25 Years of Land Cover Change
Source: PLoS One. 2013 Jan 17;8(1):e54179. doi: 10.1371/journal.pone.0054179 (PMC3547884; doi:10.1371/journal.pone.0054179)
Supplement: Table S1 — The number of sites occupied by bird species in 1982 and 2007 (N = 256). (DOC) [file pone.0054179.s002.doc]

| **Common names** | **Latin names** | **Number of sites occupied in 1982** | **Number of sites occupied in 2007** | **D²_CQO_** | **D²_GLM_** | **AUC_CQO_** | **AUC_GLM_** |
| --- | --- | --- | --- | --- | --- | --- | --- |
| **Skylark** | ***Alauda arvensis*** | 70 | 56 | 0.22 | 0.21 | 0.71 | 0.69 |
| **Tree Pipit** | ***Anthus trivialis*** | 63 | 12 | 0.09 | 0.09 | 0.66 | 0.71 |
| **Common Linnet** | ***Carduelis cannabina*** | 10 | 8 | 0.15 | 0.12 | 0.54 | 0.73 |
| **Short-toed Treecreeper** | ***Certhia brachydactyla*** | 51 | 63 | 0.08 | 0.08 | 0.74 | 0.73 |
| **Common Wood Pigeon** | ***Columba palumbus*** | 9 | 23 | 0.23 | 0.20 | 0.79 | 0.78 |
| **Carrion Crow** | ***Corvus corone*** | 12 | 6 | 0.18 | 0.15 | 0.59 | 0.51 |
| **Common Cuckoo** | ***Cuculus canorus*** | 22 | 19 | 0.07 | 0.07 | 0.84 | 0.85 |
| **Great Spotted Woodpecker** | ***Dendrocopos major*** | 15 | 22 | 0.15 | 0.15 | 0.81 | 0.81 |
| **Corn bunting** | ***Emberiza calendra*** | 51 | 45 | 0.21 | 0.21 | 0.56 | 0.60 |
| **Cirl Bunting** | ***Emberiza cirlus*** | 67 | 42 | 0.14 | 0.14 | 0.67 | 0.70 |
| **Yellowhammer** | ***Emberiza citrinella*** | 112 | 52 | 0.14 | 0.14 | 0.69 | 0.72 |
| **European Robin** | ***Erithacus rubecula*** | 107 | 88 | 0.17 | 0.17 | 0.88 | 0.87 |
| **Common Chaffinch** | ***Fringilla coelebs*** | 55 | 78 | 0.26 | 0.26 | 0.82 | 0.81 |
| **Eurasian Jay** | ***Garrulus glandarius*** | 33 | 19 | 0.11 | 0.08 | 0.73 | 0.76 |
| **Melodious Warbler** | ***Hyppolais polyglotta*** | 32 | 33 | 0.05 | 0.05 | 0.64 | 0.68 |
| **Red-backed Shrike** | ***Lanius collurio*** | 20 | 17 | 0.08 | 0.08 | 0.71 | 0.71 |
| **Woodlark** | ***Lullula arborea*** | 12 | 10 | 0.16 | 0.16 | 0.54 | 0.62 |
| **Common Nightingale** | ***Luscinia megarhinchos*** | 57 | 85 | 0.13 | 0.13 | 0.74 | 0.75 |
| **Eurasian Golden Oriole** | ***Oriolus oriolus*** | 28 | 28 | 0.14 | 0.13 | 0.81 | 0.80 |
| **Eurasian Blue Tit** | ***Parus caeruleus*** | 118 | 71 | 0.12 | 0.12 | 0.72 | 0.73 |
| **Great Tit** | ***Parus major*** | 132 | 131 | 0.06 | 0.06 | 0.72 | 0.70 |
| **Western Bonelli's Warbler** | ***Phylloscopus bonelli*** | 23 | 9 | 0.21 | 0.19 | 0.62 | 0.63 |
| **Common Chiffchaff** | ***Phylloscopus collybita*** | 76 | 85 | 0.39 | 0.35 | 0.83 | 0.86 |
| **European Green Woodpecker** | ***Picus viridis*** | 20 | 3 | 0.03 | 0.03 | 0.56 | 0.88 |
| **Common Firecrest** | ***Regulus ignicapilla*** | 32 | 8 | 0.34 | 0.34 | 0.83 | 0.85 |
| **European Stonechat** | ***Saxicola torquata*** | 64 | 43 | 0.14 | 0.14 | 0.67 | 0.67 |
| **Eurasian Nuthatch** | ***Sitta europea*** | 10 | 13 | 0.31 | 0.28 | 0.88 | 0.84 |
| **European Turtle Dove** | ***Streptopelia turtur*** | 22 | 36 | 0.09 | 0.07 | 0.51 | 0.56 |
| **Eurasian Blackcap** | ***Sylvia atricapilla*** | 192 | 187 | 0.23 | 0.23 | 0.69 | 0.70 |
| **Garden Warbler** | ***Sylvia borin*** | 19 | 3 | 0.22 | 0.22 | 0.85 | 0.86 |
| **Whitethroat** | ***Sylvia communis*** | 105 | 64 | 0.23 | 0.22 | 0.68 | 0.72 |
| **Eurasian Wren** | ***Troglodytes troglodytes*** | 136 | 83 | 0.11 | 0.11 | 0.62 | 0.64 |
| **Common Blackbird** | ***Turdus merula*** | 145 | 103 | 0.12 | 0.12 | 0.68 | 0.68 |
| **Song Thrush** | ***Turdus philomelos*** | 36 | 32 | 0.35 | 0.35 | 0.75 | 0.80 |
| **Eurasian Hoopoe** | ***Upupa epops*** | 6 | 7 | 0.29 | 0.25 | 0.62 | 0.74 |
